# Improved appreciation of the functioning and importance of biological soil crusts in Europe: the Soil Crust International Project (SCIN)

**DOI:** 10.1007/s10531-014-0645-2

**Published:** 2014-03-02

**Authors:** Burkhard Büdel, Claudia Colesie, T. G. Allan Green, Martin Grube, Roberto Lázaro Suau, Katharina Loewen-Schneider, Stefanie Maier, Thomas Peer, Ana Pintado, José Raggio, Ulrike Ruprecht, Leopoldo G. Sancho, Burkhard Schroeter, Roman Türk, Bettina Weber, Mats Wedin, Martin Westberg, Laura Williams, Lingjuan Zheng

**Affiliations:** 1 Plant Ecology and Systematics, Biology, University of Kaiserslautern, Erwin-Schrödinger-Str. 13, 67663 Kaiserslautern, Germany; 2 Departamento de Biología Vegetal II, Facultad de Farmacia, Universidad Complutense de Madrid, 28040 Madrid, Spain; 3 Biological Sciences, University of Waikato, Private Bag 3105, Hamilton, New Zealand; 4 Institute of Plant Sciences, University of Graz, Holteigasse 6, 8010 Graz, Austria; 5 Arid Zones Research Station (CSIC), Carretera Sacramento, s/n 04120 –La Cañada de San Urbano, Almeria, Spain; 6 Department of Organismic Biology, University of Salzburg, Hellbrunnerstr. 34, 5020 Salzburg, Austria; 7 Botanical Institute and Botanical Gardens, Plant Ecophysiology, University of Kiel, Am Botanischen Garten 1-9, 24118 Kiel, Germany; 8 Multiphase Chemistry Department, Max-Plank Institute for Chemistry, Hahn-Meitner-Weg 1, 55128 Mainz, Germany; 9 Department of Botany, Swedish Museum of Natural History, P.O. Box 50007, 10405 Stockholm, Sweden

**Keywords:** Biological soil crust, Net primary productivity, Biodiversity, Soil microorganisms, Lichens, Bryophytes

## Abstract

Here we report details of the European research initiative “Soil Crust International” (SCIN) focusing on the biodiversity of biological soil crusts (BSC, composed of bacteria, algae, lichens, and bryophytes) and on functional aspects in their specific environment. Known as the so-called “colored soil lichen community” (Bunte Erdflechtengesellschaft), these BSCs occur all over Europe, extending into subtropical and arid regions. Our goal is to study the uniqueness of these BSCs on the regional scale and investigate how this community can cope with large macroclimatic differences. One of the major aims of this project is to develop biodiversity conservation and sustainable management strategies for European BSCs. To achieve this, we established a latitudinal transect from the Great Alvar of Öland, Sweden in the north over Gössenheim, Central Germany and Hochtor in the Hohe Tauern National Park, Austria down to the badlands of Tabernas, Spain in the south. The transect stretches over 20° latitude and 2,300 m in altitude, including natural (Hochtor, Tabernas) and semi-natural sites that require maintenance such as by grazing activities (Öland, Gössenheim). At all four sites BSC coverage exceeded 30 % of the referring landscape, with the alpine site (Hochtor) reaching the highest cyanobacterial cover and the two semi-natural sites (Öland, Gössenheim) the highest bryophyte cover. Although BSCs of the four European sites share a common set of bacteria, algae (including cyanobacteria) lichens and bryophytes, first results indicate not only climate specific additions of species, but also genetic/phenotypic uniqueness of species between the four sites. While macroclimatic conditions are rather different, microclimatic conditions and partly soil properties seem fairly homogeneous between the four sites, with the exception of water availability. Continuous activity monitoring of photosystem II revealed the BSCs of the Spanish site as the least active in terms of photosynthetic active periods.

**Electronic supplementary material:**

The online version of this article (doi:10.1007/s10531-014-0645-2) contains supplementary material, which is available to authorized users.

## Introduction

Bare ground is not just abiotic ground; in fact, the soil surface in areas free of higher vegetation is often covered by a skin made up of a community of microorganisms, like cyanobacteria, algae, lichens and bryophytes—forming a complex structure known as biological soil crust (BSC). Biological soil crusts can be the only vegetation cover in arid and semi-arid regions such as hot and cold deserts or xerothermic steppe vegetation (Belnap and Lange 2003). They are also the first colonizers of disturbed soils and have major impacts on the soil properties through stabilization, erosion limitation, and facilitation of colonization by higher plants (Malam 1998; Belnap et al. 2003b; Thomas and Dougill 2007; Guo et al. 2008).

Despite these immensely important properties, soil crusts are neither well understood nor well appreciated by conservation and regulation authorities who are missing opportunities for improved policies and actions in the area of land protection. Yet they are the natural and most effective force in land stabilization and recovery (Campbell 1979; Campbell et al. 1989; Belnap et al. 2003a).

While the dynamics and ecology of BSCs in arid and semiarid regions of the world are well documented over the last decade (Belnap and Lange 2003; Maestre et al. 2011; Pointing and Belnap 2012), investigations in temperate regions have mainly focused on floristic and phytosociology, rather than functional aspects (Büdel 2003). From these studies it is known that the “Bunte Erdflechtengesellschaft” (colored soil lichen community; Reimers 1950, 1951), composed of communities of the Fulgensietum fulgentis and Cladonietum symphycarpae complex, has a wide distribution ranging from the southern Swedish Alvar region in the north (Bengtsson et al. 1988; Albertson 1950) to southern Algeria, and from the Poitou and the Eifel midlands in the west to the Aralo-Caspian semideserts and the Mesopotamian region in the east (Müller 1965). The presence of this arid microclimate-adapted (Hahn et al. 1989; Lange et al. 1995) community of colored soil lichens, centered in the Mediterranean and the continental areas of the Eurasian continent, may be explained as a relic of the postglacial warm period (Reimers 1940). In Western Europe, the existence of the colored soil lichen community is restricted to sites largely free of vascular plant vegetation, sites that can either originate from human impact or from environmental conditions. Extreme dryness, hot or cold temperatures or long lasting snow cover can restrict higher plant growth and therefore provide natural environments suitable for BSC development. On the other hand, soil and plant removal, for strategic reasons as for example in front of medieval castles, or heavy grazing can also restrict higher plants and provide human influenced environments ready for colonization with BSCs. As these areas are no longer managed, these unique BSC communities are endangered, several attempts to protect them have been made by national nature conservation authorities (e.g. in Bavaria, Germany; Dunkel 2003).

**Fig. 1:**
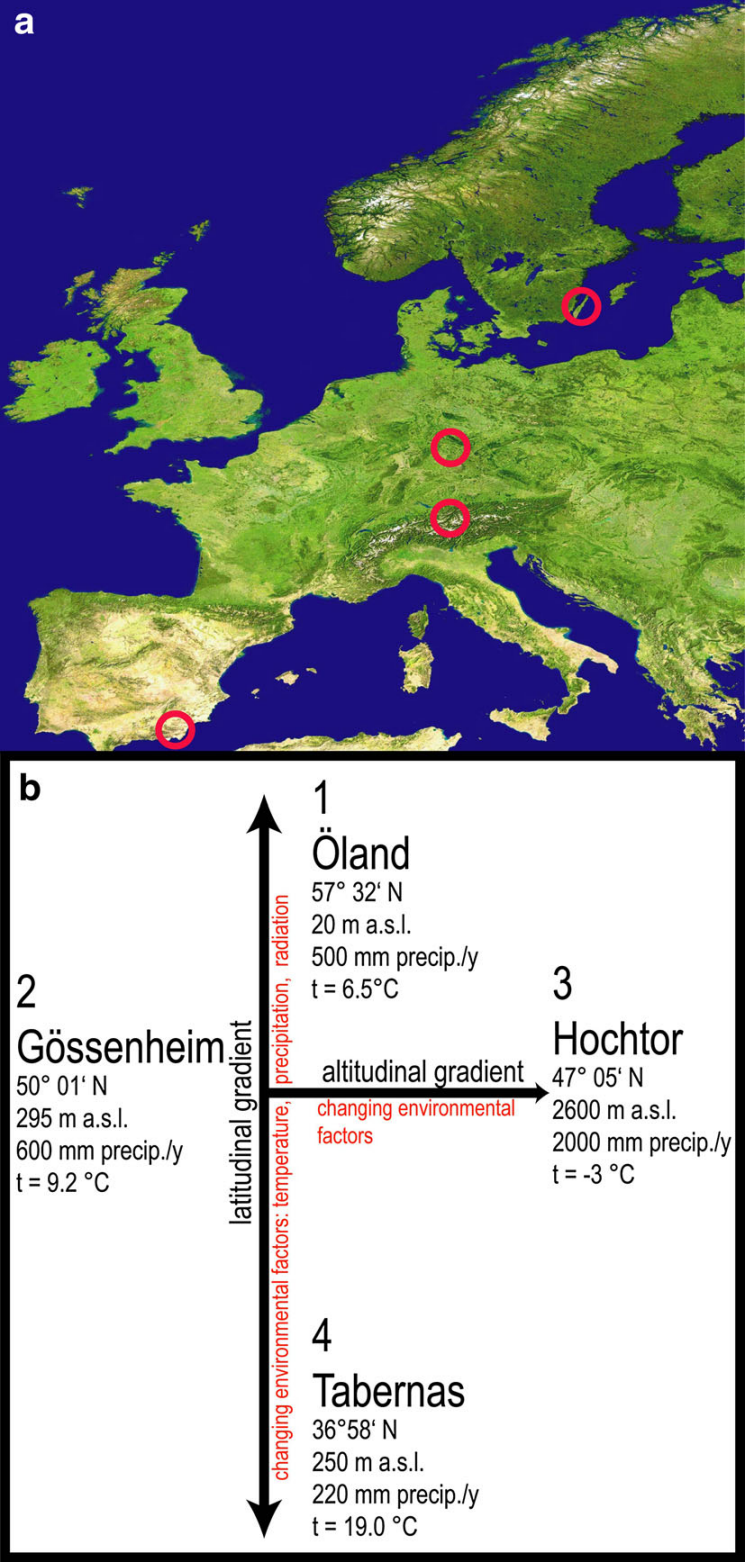
**a** Map of investigation sites (*red circles*) in Western Europe (© USGS). **b** Latitudinal and altitudinal gradient of the investigation sites with basic data

**Fig. 2:**
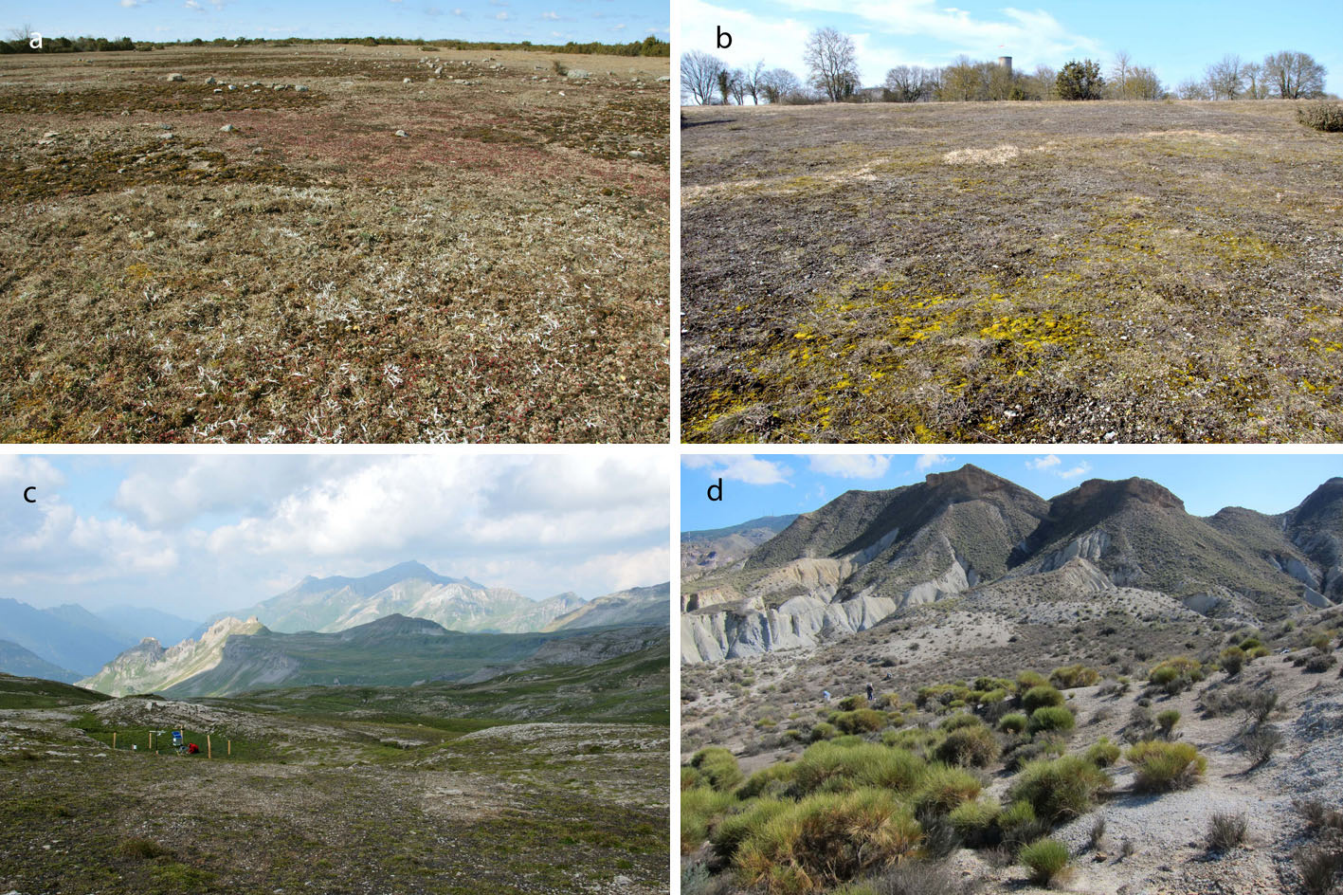
Investigation sites; **a** in the Gynge Alvar at Öland, Sweden; **b** in the xerothermic steppe vegetation, nature reserve “Ruine Homburg”, near Gössenheim, Germany; **c** Hochtor near the Großglockner High Alpine Road, Austria; **d** Tabernas Badlands, near Almeria, Spain

Initiated by the 2010–2011 joint call of BiodivERsA European network “Valuation of biodiversity and ecosystem services, and better incorporation of biodiversity and ecosystem services into society and policy” (see http://www.biodiversa.org/79), we launched a project on European BSCs to answer these questions. We established an international research project along a 20° latitudinal and a 2,300 m altitudinal gradient, extending from the Gynge Alvaret at Öland, Sweden through the xerothermic steppe vegetation at Gössenheim, Germany, up to the Hochtor at 2,600 m in the Großglockner Massif of the Alps, Austria, and to the southernmost locality, the Tabernas badlands north of Almeria, Spain (Figs. 1a, b, 2a–d).

As all four sites are located at different macro climatic regions, the question arises whether it is basically microclimate that determines the community structure and function, human impact or a mixture of both. From this several questions can be inferred: (1) How large is the inter- and intraspecific variability of the BSC communities between different sites? (2) To what extent is adaptation/acclimation responsible for the wide distribution range of the characteristic species? (3) How can these communities be protected?

The aim of our international research project, the details of which are presented here, is to provide a much improved understanding of BSC functionality from the desert, to the alpine ecosystems. Functional studies are backed by detailed biodiversity assessments that aim to reveal the key organisms that influence BSC functioning over this wide latitudinal, altitudinal and climatic range. Information transfer to stakeholders is achieved through a series of consultations and reports including highly visual material supporting their work. We intend to achieve all of this using a structure with different work packages (WP) performing the research and data gathering, which are coordinated by the scientific oversight committee with members of each WP plus an external expert scientist of the research field (supplementary material Fig. 1).

In the different WPs we encompass the specific habitat properties of all sites such as the meso- and microclimate, soil properties, water availability, and human impact. As variables, we determine BSC coverage, the BSC-type diversity, the BSC species composition and diversity, as well as activity and biomass of the BSCs.

In WP 1 we aim to close the biodiversity gap for European BSCs investigating non-photosynthetic bacteria with molecular techniques, cyanobacteria, lichens and fungi in a polyphasic approach (molecular and classical), and bryophytes by classical morphology based techniques. In WP 2 the annual net carbon gain of typical BSCs at the four investigation sites will be obtained from a model linking three sets of measurements: chlorophyll fluorescence monitoring of activity, continuous CO_2_-gas exchange measurements of BSCs in the field, and CO_2_-gas exchange response curves of typical BSCs under controlled conditions. Assessing soil properties, structure and soil hydrology as influenced by the presence of BSCs is the aim of WP 3. To achieve this, at each site, soil types are described and soil samples are taken from different strata, including crust layer and underlying soil. Within WP 4 we are quantifying community structures, BSC coverage and biomass and the ability to recover from vegetation removal. In WP 5 the degree of adaptation, acclimation and uniqueness of the key BSC species is assessed by measuring their genetic and morphological diversity and their eco-physiological properties. Adaptation/acclimation will also be measured by cross transplantation of major lichens from and to each site. Another research focus will be whether the lichens have photobiont populations that are different within the same lichen species and also geographically. An increasing number of scientific publications show, that chlorolichens use local populations of green algae as photobionts, while cyanobacterial lichens seem to preferably select highly efficient cyanobiont strains, which are shared by ecologically similar lichenized fungi (Printzen et al. 2010; Fernández-Mendoza et al. 2011). Finally WP 6 ensures the coordination and successful delivery of material with end-users. This WP performs the important functions of overseeing both the science part of the project and providing the link with the stakeholders. For this reason the WP team is composed of the leaders of the other packages, although others will naturally be involved, and a science education specialist. The scientific outputs shall be changed into a form that is more easily understood by stakeholders and end-users, and most importantly, assure the awareness and appreciation of BSCs as an important component of the landscape (see also homepage of the project at http://www.soil-crust-international.org/).

## Materials and methods

### Investigation sites

1. Nature Reserve Gynge Alvar, Öland, Sweden (Fig. 2a). The site (56°32′′N, 16°28′E) is situated in Mörbylånga comunity, Resmo parish, about 20 m above sea level (a.s.l.), on the island of Öland, Sweden. Öland has a maritime climate, but is situated in a rain shadow and, with 500 mm/year, has the lowest mean precipitation of any Swedish provinces. The mean temperature is about −2 °C in February and 17 °C in July (annual mean 1961–1990). Gynge Alvar Nature Reserve is part of the ca. 26,000 ha large Stora Alvaret (the Great Alvar) which together with other agricultural areas on southern Öland is designated as a World Heritage Site by UNESCO. The site at Gynge Alvar is a typical open limestone pavement alvar area, with Ordovician sedimentary limestone as bedrock and a very thin layer of gravel and scattered siliceous moraine rocks. It is currently grazed by cattle. On the open soil-crust dominated areas higher plants are scarce and the cryptogam vegetation is dominated by lichens such as *Cladonia symphycarpia*, *C. rangiformis,* *C. foliacea*, *Thamnolia vermicularis,* *Squamarina cartilaginea*, *Fulgensia bracteata*, *Fulgensia fulgens*, *Psora decipiens*, and cyanobacteria (Albertson 1950; Fröberg 1999). The alvar regions are usually seen as semi-natural open areas on limestone pavement which have existed since the last glaciation (ca 11,000 years before present), containing both relicts from postglacial arctic conditions and from later steppe-like conditions in warm periods. These areas were thus originally open and dependent on grazing from larger herbivores to remain so. Later human settlers have continued the grazing activities with cattle, horses and sheep. It is clear that at least those areas with somewhat thicker soils will become overgrown by shrubs if grazing stops. The alvar areas, therefore, result from a combination of naturally thin soils on limestone pavement bedrock, grazing by larger mammals, and continuous human impact for thousands of years, particularly through livestock grazing regimes and removal of firewood.
2. Nature Reserve “Ruine Homburg” at Gössenheim, northern Bavaria, Germany (Fig. 2b). The site is situated at 50°01′N and 9°48′E in an area with Triassic shell limestone (Muschelkalk) as bedrock. The elevation is 295 m a.s.l. The climate is warm temperate; mean air temperature in January is −0.3 °C and 18.3 °C in July (annual mean 9.2 °C). Annual precipitation is 600 mm. The vegetation is composed of a relic flora, together with sub-Mediterranean-continental (*Carex humilis*) and sub-Mediterranean-sub-atlantic (*Trinia glauca*) elements (Lösch 1981). It is an open anthropogenic landscape with bare rock and gravel spots covered by a thin vegetation layer dominated by cryptogams of the association Toninio-Psoretum decipientis in the class Psoreta decipientis (*Collema tenax, Cladonia convoluta* (=*C. folicaea* according to Pino-Bodas et al. (2010))*, F. fulgens, P. decipiens, Squamarina lentigera, Toninia sedifolia*, as well as a number of cyanobacteria and bryophytes (Hahn et al. 1989; Lange et al. 1995; Büdel 2003). The nearby castle was founded in 1080 and is the reason that the landscape has remained open.
3. Hochtor, near the Großglockner High Alpine Road, Hohe Tauern National Park, Austria (Fig. 2c). The site is situated in the high mountains of Hohe Tauern (Austria), close to the Grossglockner High Alpine Road at 47°05′ N and 12°51′ E. The area is part of the upper Schieferhülle (Tauern window); in the stricter sense it belongs to the Seidlwinkl Triassic, which mostly consists of lime marble, dolomite and Rauwacke. The elevation ranges from 2,500 to 2,600 m a.s.l. The climate is alpine; mean air temperature is around −10 to −8 °C in January and 2–4 °C in July. On average, there are 250 frost days, 150–200 ice days and 80–90 frost alternation days each year. Mean annual precipitation is between 1,750 and 2,000 mm, with more than 70 % as snow. Snow cover lasts for 270–300 days. Under these climatic conditions development of soil and the subsequent establishment of higher plants is extremely slow; Skeletic Regosols and Rendzic Regosols on fine weathered carbonatic (gypsiferous) material prevail. Typical lichens are *F. bracteata*, *P. decipiens*, *Toninia diffracta*, *T. vermicularis* and many others, together with cyanobacteria, green algae and bryophytes (Peer et al. 2010). Vascular plants, small cushion plants, creeper and tuff grasses occur whereas bryophytes are rare.
4. Tabernas field site, north of Almeria, Spain (Fig. 2d). The site (37°00′N, 2°26′W) is located in the Tabernas basin, surrounded by the Betic Cordilleras and subsequently filled by Serravallian—early Messinian continental and marine sediments. The parent material is a gypsum-calcareous mudrock mainly composed by silt-size (>60 %) siliceous and gypsum-calcareous particles. The climate of the area is semi-arid warm-Mediterranean, with a mean annual precipitation of 220 mm (with 37 % of inter-annual variation and 76 to 215 % of monthly variation). The number of days with rain each year varies from 25 to 55 (average 37). Mean annual temperature is 18.5 °C, with a monthly mean of 4.1 °C in the coldest month and 34.7 °C in the hottest month. Potential evapotranspiration is around 5–7 times higher than annual precipitation. The average annual insolation is more than 3,000 h/year.
  About one-third of the total badland surface consists of eroded soil which is almost bare; another third is covered by a mosaic of grasses, shrubs, annual plants and BSCs, often dominated by lichens. The remaining third is mainly covered by BSC, with some sparse vascular plants. Shrubs include several endemics and a high proportion of Iberian-North African species. BSCs include cyanobacteria, occasional mosses and numerous lichens (*Catapyrenium rufescens, Cladonia convoluta, Collema cristatum, Diplochistes diacapsis, Endocarpon pusillum, Fulgensia fulgida, F. poeltii, F. desertorum, Placynthium nigrum, Psora albilabra, P. decipiens, Squamarina cartilaginea, T. sedifolia*, etc.) (Gutiérrez and Casares 1994). Land use has probably been minimal during the last 60 years and certainly it has been very light during the last 23 years. The area has been protected since 1989 as “Paraje Natural”.

### Methods

#### Climate

All investigation sites are equipped with similar climate stations, monitoring wind speed and direction, air temperature, air humidity, solar radiation (Photosynthetically Active Photon Flux Density, PPFD), UV-radiation, and precipitation every 5 min (supplementary material Fig. 2a). All stations run for at least one year, but preferably 2–2.5 years. Where necessary, the climate stations are fenced as security against damage.

#### Vegetation analyses

Sampling for the vegetation analyses, biodiversity and soil property assessment was conducted in one concerted approach: First, at each of the four geographical sites, homogeneous vegetation units 100 × 100 m were defined and coverage of the different elements was determined by 150 subplots 25 × 25 cm applying the point-intercept method. We differentiated between BSCs light and BSCs dark, the latter represent successional development of BSC from a species-poor, light-coloured cyanobacterial BSC to a species-rich BSC community dominated by dark cyanobacteria (Belnap and Eldridge 2003), cyanolichen-dominated, chlorolichen-dominated, bryophyte-dominated, vascular plants, litter, open soil, stones and gravel.

Second, 10 restoration plots were established at each of the four geographical sites in relatively well-developed vegetation units to investigate the speed and successional pattern of BSC recovery. Each restoration plot (100 × 100 cm) is accompanied by a control plot (100 × 100 cm; supplementary material Fig. 2b). Each restoration plot was cleared of BSC (removal of the upper 1–4 cm) and then checked for recovery every 6 months by measuring surface hardness, chlorophyll content, chemical and physical soil parameters, and identification of new established species.

#### Soils

The physico-chemical properties and hydrological parameters of crust and underlying soil from four sites were analyzed. The pH of soil from 5 to 10 cm underneath the crust and directly from the crust (~3–5 cm^2^) was determined in 0.01 M CaCl_2_ solutions; electrical conductivity in 1:5 soil–water suspensions (Visconti et al. 2010), when the pH values of the soil samples was above 7, we used 0.1 M triethanolamine–buffered BaCl_2_ solution to extract K, Ca, Na and Mg. For particle size distribution two methods were used: the sieving and pipette method (ÖNORM L 1061, 1988), for particle size distribution analysis soils were dispersed in 0.1 mol/l Na_4_P_2_O_7_ solution overnight prior to the sieving process; water holding capacity by gravimetric after soil saturation with water and drying at 105 °C (Wilke 2005); aggregate stability by modified wet sieving method (Kværnø and Øygarden 2006); exchangeable K, Ca, Na and Mg in 0.1 mol/l BaCl_2_ extraction solution by flame atomic absorption spectrophotometry (FAAS); plant available phosphate was measured according to calcium–acetate–lactate CAL-method by Schüller (1969); water repellence by water drop penetration time test (Adams et al. 1969; Rodriguez-Caballero et al. 2013); hydraulic conductivity by mini-disc infiltration. In addition, contents of total organic C, total N, δ15 N and δ13C in crust and underlying soil are measured by elemental analyzer-isotope ratio mass spectrometry (EA-IRMS) to provide insight into the N- and C-turnover. Values given in the text are mean ± standard deviation. The terminology of soil types used throughout the text follows the World reference base for soil resources (WRB 2006) by the FAO.

#### Diversity and community composition

Next-generation sequencing technology was used to assess the diversity and community composition of bacteria and fungi. Collected samples were immediately placed on dry ice and stored at −70 °C until DNA extraction with the PowerSoil^®^ DNA Isolation Kit (MO BIO, Carlsbad, CA). DNA was subjected to 16S rRNA gene amplicon pyrosequencing (Roche 454 FLX Titanium) using primers targeting the bacterial V4 hypervariable region (Bates et al. 2011). For analysis of fungi, primers targeting the ITS region were used. 454 sequence data were processed using the default workflow in QIIME v. 1.6.0. (Caporaso et al. 2010). To localize microorganisms in BSCs, we used light and confocal laser scanning microscopes (CLSM) in conjunction with fluorescence in situ hybridization (FISH) technique. DNA-Extractions and the fingerprinting method DGGE for 16S rDNA-gene (Nübel et al. 1997) were used to determine the taxonomic composition and genetic variation of Cyanobacteria within the BSCs. Genetic identification of green algal photobionts (chlorobionts) was carried out using the nuclear marker nrITS and the chloroplast marker psbJ-L (Ruprecht et al. (2014).

#### Taxonomic diversity assessment and phylogenetic species delimitation studies

Lichens were identified using appropriate identification keys for the different countries (e.g. Smith et al. 2009; Wirth et al. 2013a; 2013b), and in many cases aided by comparison with original taxonomic literature and verified voucher specimens. In several groups, species delimitation studies are conducted using multi-gene phylogenies. The moss species were determined by experts on the local flora and names are according to Hill et al. (2006) and Köckinger et al. (2013). Cyanobacteria and algae were identified by light microscopy of soil samples and appropriate taxonomic keys (Geitler 1932; Komárek and Anagnostidis 1998; 2005; Ettl and Gärtner 1995).

#### Morphology

Thallus size (n = 30, independent individuals) was determined and layer thicknesses (upper cortex, photobiont layer, medulla, lower cortex (where present) were measured on freezing microtome sections (n = 300 from 30 independent thalli) for selected key lichen species.

#### Net carbon gain

A model linking 3 sets of measurements was used to calculate net carbon gain: (1) Chlorophyll fluorescence monitoring of activity (supplementary material Fig. 2c–e), at least one year of data from each site (2 preferred) is obtained by using a chlorophyll fluorescence based device measuring the yield ((Y = Fm′−F)/Fm′, with F being the basal fluorescence and Fm′ the maximal fluorescence following a saturation pulse) of PS II (MONI-DA, Gademann Instruments, Würzburg). (2) CO_2_-exchange of BSCs in the field using a portable gas exchange fluorescence system (GFS-3000, Walz, Effeltrich), acquiring at least 14 days of continuous records from each site. (3) The response of net CO_2_-exchange of BSCs to environmental factors in the lab under controlled conditions. Particular attention is given to lichenized fungal species and cyanobacteria, which are key ecological components of soil crusts. Values given in the text are mean ± standard deviation.

#### Adaptation/acclimation/genetic uniqueness of key organisms

Lichens of the same species from all four sites were sampled to test whether they show the same CO_2_-exchange behavior, a climate-specific acclimation and whether they have local photobiont populations. Five to ten subpopulations of selected lichen species were sampled from each site. Genetic variation is investigated by haplotype identity using DNA sequences from both mycobionts and photobionts, this data will be correlated with measurements of morphological traits such as surface area and thallus thickness, and also related to CO_2_-exchange data.

#### Transplantation

The following species are transplanted from every site to all other sites and will be analyzed for changes in morphology, photosynthetic performance and their photobionts after 1.5 years: *P. decipiens*, *T. sedifolia*, *Peltigera rufescens*, *F. fulgens*, *F. bracteata*, and *Diploschistes muscorum*.

## Results and discussion

### Vegetation analysis, coverage, biomass

**Fig. 3:**
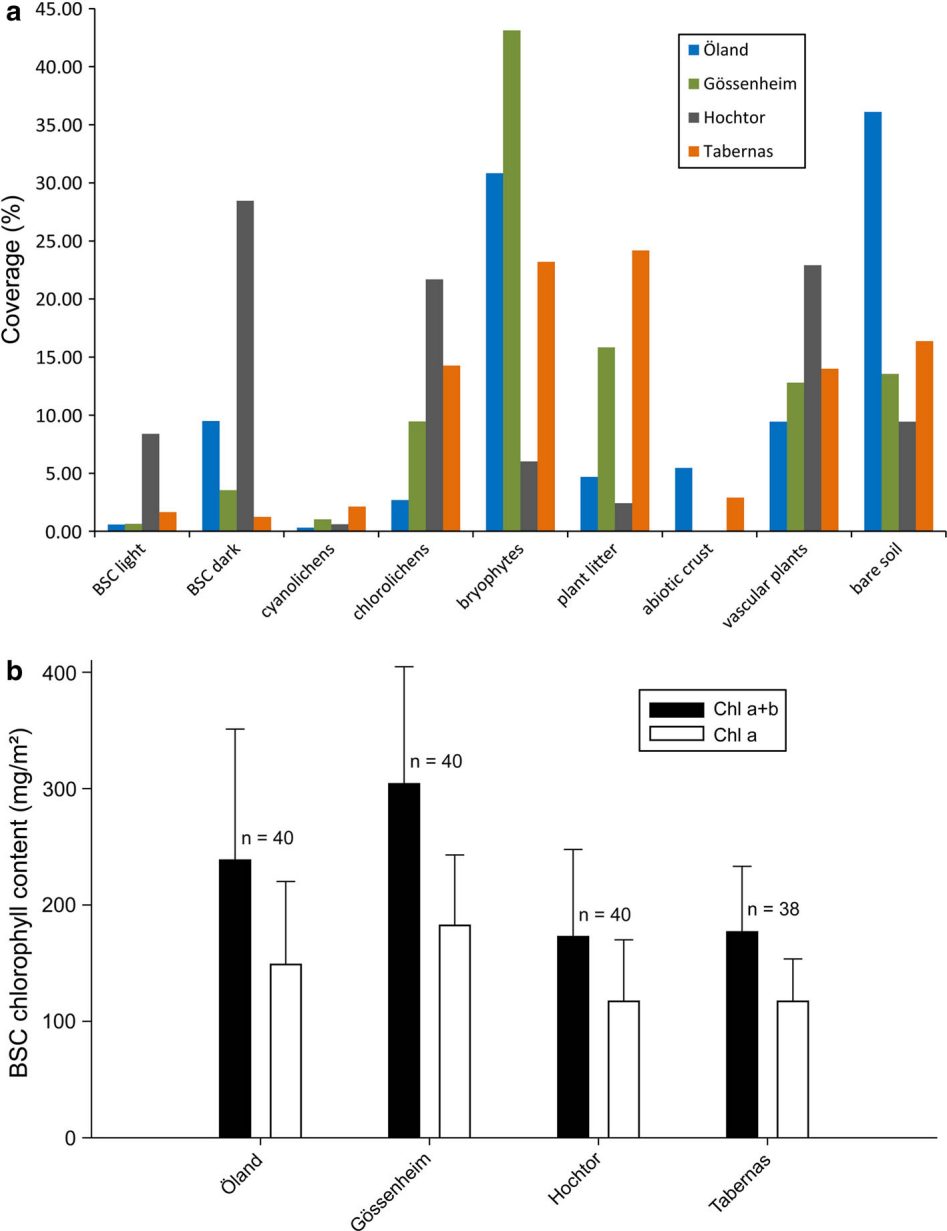
**a** Coverage of the different crust types and other vegetation at all sites; **b** chlorophyll content (*a* and *a* + *b*; lines in bars show standard deviation) at all sites

The analyses of the different crust types coverage (BSC light, BSC dark, cyanolichens, chlorolichens, bryophytes, plant litter, abiotic crust, vascular plants, and bare soil) revealed, that the Hochtor site has by far the highest coverage of cyanobacteria (BSC light and BSC dark), chlorolichens and also a high proportion of vascular plants (Fig. 6a, g). Bryophytes (mainly mosses) dominate at the Gössenheim, Öland and Tabernas sites, with Gössenheim having the highest moss coverage of more than 40 % (Fig. 3a). At these three sites, cyanolichen coverage is well below 5 % and the amount of the bare soil fraction is highest at the Swedish Öland site, followed by the Tabernas site (Fig. 6a).

Biological soil crust chlorophyll *a* and chlorophyll *a* + *b* content reached values around 200 mg chlorophyll *a* + *b* per m^2^ at all sites with slightly higher values at the human influenced sites Öland and Gössenheim (Fig. 3b). This places the four SCIN-BSC sites at the lower end of the soil crust chlorophyll *a* + *b* content scale, ranging from 980 mg/m^2^ in the local steppe formation near Würzburg, Germany to 500 mg/m^2^ in the Namib Desert, Namibia and down to 380 mg/m^2^ in Utah, USA (Lange 2003). However, the SCIN-BSC values are comparable to those of the BSCs found along the BIOTA-South transect in South Africa and Namibia (Büdel et al. 2009).

### Soil properties and structure

Soil types at the Öland site are skeletal and Rendzic Leptosols with a depth of less than 20 cm and Ai, (B), BC, and C horizons. The bedrock is an Ordovician limestone with “alvarmo layers” (cromic, relic?). Soil pH is 7.35 ± 0.05 (n = 40), while the pH of the BSC is 7.3 ± 0.06 (n = 40). At the Gössenheim site, soil types are skeletal, Rendzic Leptosols with a depth of less than 10 cm and AC and C horizons. The bedrock is a Triassic shell limestone (Muschelkalk) with characteristic top soil removal. Soil pH is 7.37 ± 0.06 (n = 40), while the pH of the BSC is 7.33 ± 0.07 (n = 40). Soil types at the Hochtor site are calcareous Regosols and Rendzic Leptosols with a depth of 15–30 (>50) cm and A1, A2, C1, and C2 horizons, with a buried iron-humus layer. The bedrock is Triassic Seidlwinkl and Rauwacke. Soil pH is 7.43 ± 0.09 (n = 40), while the pH of the BSC is 7.34 ± 0.05 (n = 40). Soil types at the Tabernas site are Haplic Calcisols with a depth of less than 100 cm and A, AC, Ck1, Ck2, and C3 horizons, originating from Miocene sediments (gypsum-calcitic mudstone and sandstones) with a surface accumulation of gypsum. Soil pH is 7.4 ± 0.06 (n = 40), while the pH of the BSC is 7.03 ± 0.1 (n = 40).

**Fig. 4:**
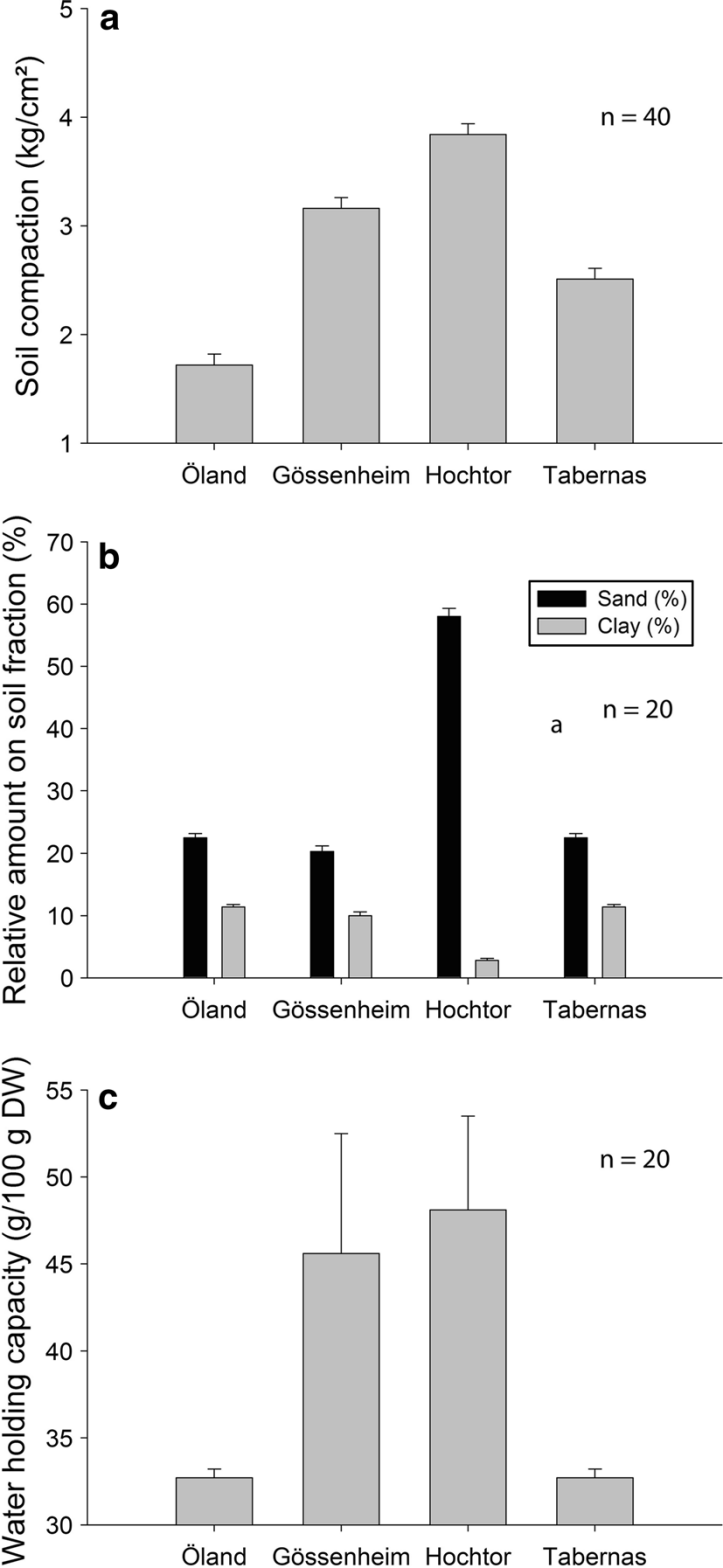
Soil characteristics at all four sites: **a** soil compaction; **b** soil fractions; **c** water holding capacity of soils (*lines* in bars show standard deviation)

Soil compaction was highest (3.84 ± 0.1 kg/cm^2^) and clay content lowest (<3 %) at the Hochtor site (Fig. 4a–b) which also had the highest water holding capacity, 48.1 ± 5.4 g H_2_O/100 g dry weight (Fig. 4c). At all sites the water holding capacity of the BSC was significantly higher than in the underlying soils.

### Bacterial diversity

**Fig. 5:**
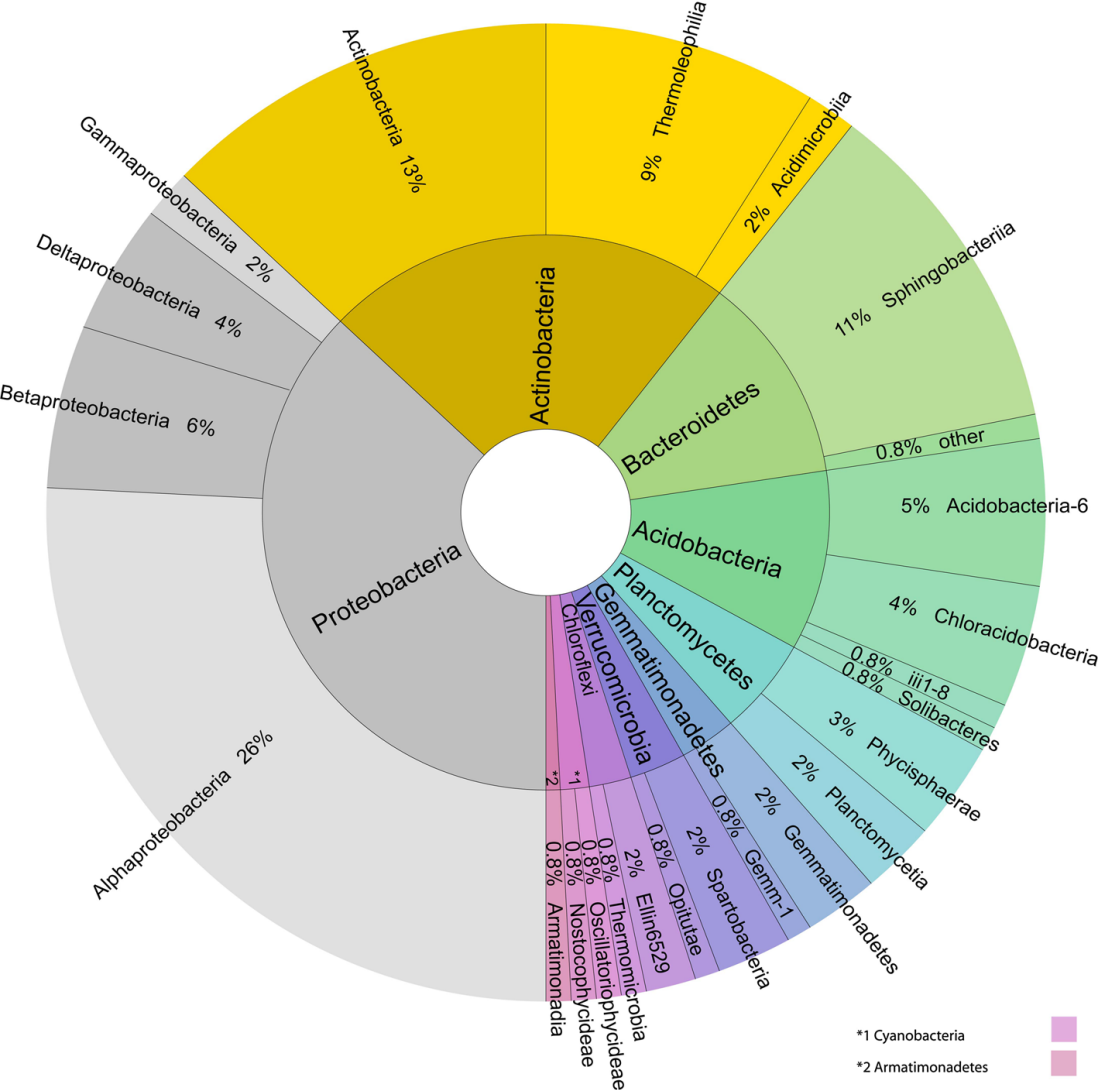
Core microbiome (125 OTUs) based on 10 samples per location processed in QIIME (sequences were denoised, assigned to OTUs at a 98 % similarity threshold, rarified to 732 reads) OTUs found at all four locations were considered part of the core

Non-photosynthetic bacteria were only quite recently considered as important BSC-organisms (Garcia-Pichel et al. 2003; Castillo-Monroy et al. 2011) and their important role in the nitrogen budget of BSCs has been addressed in several recent works (Green et al. 2008; Brankatschk et al. 2012; Barger et al. 2013). In our investigation so far, we found a shared fraction (potential core microbiome) comprising 125 operational taxonomic units (OTUs based on presence/absence data) across BSCs from the four investigation sites (Fig. 5). Relative composition analysis across the four sites revealed the Alphaproteobacteria as the dominating group, followed by the Actinobacteria (Fig. 5). The small number of shared OTUs among sites in comparison to the total number of OTUs suggests a minimal core microbiome (Maier et al. 2014).

### Cyanobacterial and green algal diversity

**Fig. 6:**
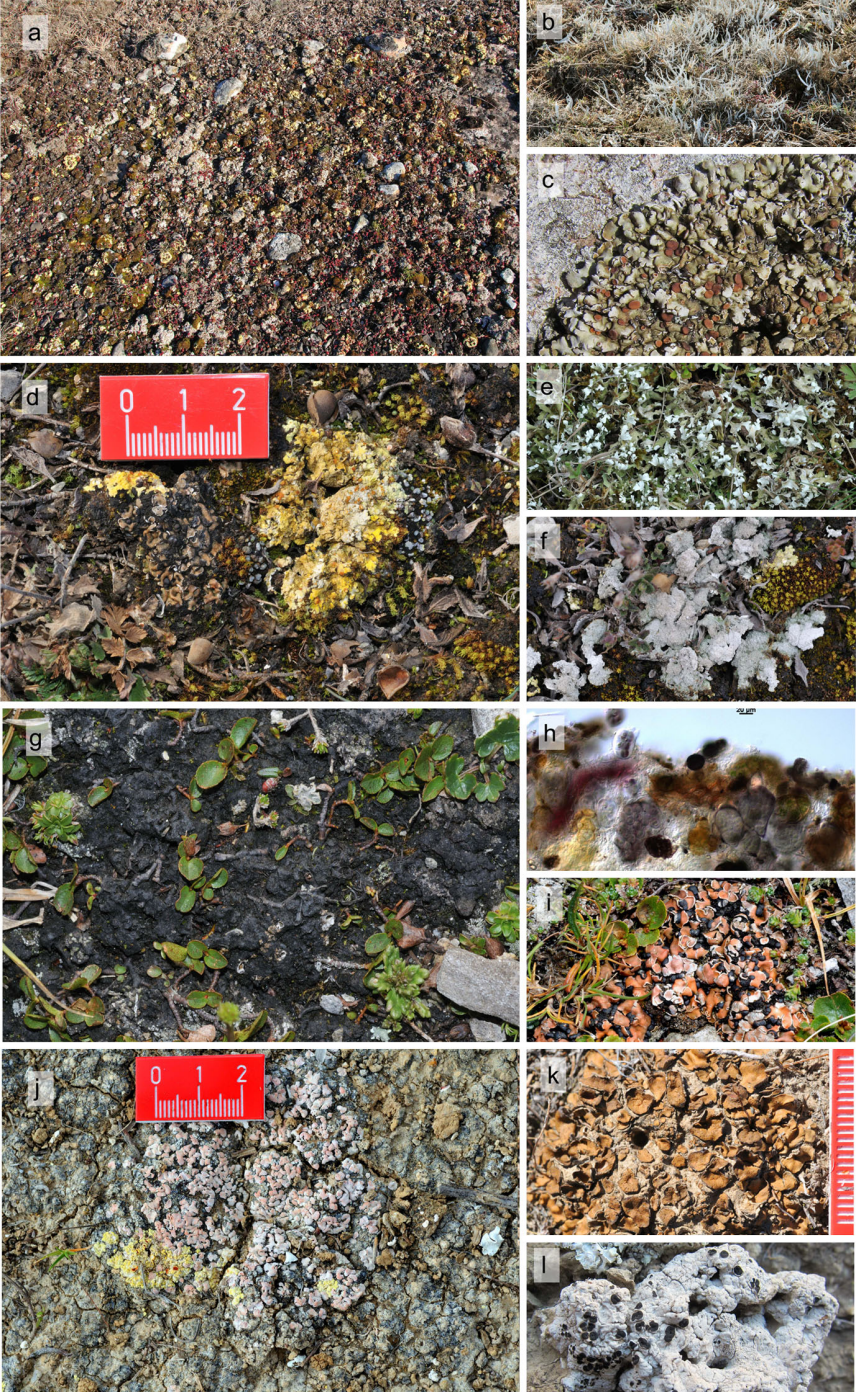
Biological soil crusts and typical lichens. **a–c** Biological soil crust at Öland; **b** the green algal lichen *Thamnolia vermicularis*, arctic-alpine lichen; **c** *Squamarina cartilaginea*, boreal to mediterranean, element of colored soil lichen community; **d–f** Gössenheim, colored soil lichen community with *Fulgensia fulgens* (yellow), *Psora decipiens* (red–brown), *Toninia sedifolia* (grey), and cyanobacteria; **e** *Cladonia foliacea*, mediterranean element, and **f** *Diploschistes muscorum*, boreal to mediterranean; **g–i** cyanobacterial crust at Hochtor; **h** cross section of the upper part of the crust, several cyanobacteria embedded in a common sheath, *Gloeocapsa* spp. (reddish + blue), *Nostoc* sp. (brown); **i** *Psora decipiens*, at all four sites, boreal to mediterranean; **j-l** biological soil crust at Tabernas, Spain with *Psora decipiens* (pink) and *Fulgensia bracteata* (yellow); **k** *Heppia despreauxii*, xeric species (scale bar unit = 1 mm); **l** *Acarospora nodulosa*, lichenicolous on *Diploschistes* species, semi-arid to arid regions of Asia, North America, Europe, Africa, and Australia

The vast majority of the bacterial diversity is non-photosynthetic bacteria. Cyanobacteria contribute only 1.6 % of the bacterial diversity (Fig. 5). Nevertheless, their contribution to biomass and especially their role in establishing BSCs is suggested to be reciprocal to their diversity (Campbell 1979; Campbell et al. 1989; Belnap et al. 2003a). To date, we have found nineteen different species/genera at all sites, with Gössenheim having the lowest number (7) compared to Hochtor (10), Öland (11) and Tabernas (13), despite the latter having the lowest coverage of light and dark BSCs. Species of the genera *Microcoleus*, the functionally most important genus in forming the initial crusts (Belnap and Gardner 1993; Malam et al. 1999) and *Nostoc*, important nitrogen fixers (Beyschlag et al. 2008; Maqubela et al. 2008), were present at all four sites. At Hochtor an extensive blackish to brown crust (Fig. 6g), often misidentified as the green algal lichen *Toniniopsis obscura* (Peer et al. 2010), was found to consist of cyanobacteria species (*Gleocapsa* spp. *Nostoc* sp. and others) with only few unicellular green algae (Fig. 6h). Peer et al. (2010) published a list of cyanobacteria and green algae found in the BSCs at the Hochtor locality based on classical morphological determination. They found six filamentous and one unicellular Cyanobacteria and 34 mostly unicellular green algal species.

The lichen photobiont green algal diversity is unexpectedly high with 12 well supported clades for *Trebouxia* spp. and 5 clades for *Asterochloris* spp. Most of the species are quite cosmopolitan, but nevertheless 5 clades are more specific and cluster according to the climatic conditions at the sampling sites (Ruprecht et al. 2014).

### Lichen diversity

The total number of lichens found for all four sites was 144 species, with the Hochtor site being the richest with 62 species (Fig. 6g–i; Table 1), followed by the Tabernas site (Fig. 6j–l; Table 1) and Öland (Fig. 6a–c; Table 1). The Gössenheim site had the lowest lichen diversity with only 25 species (Fig. 6d–f; Table 1). The highest percentage (28 %) of cyanobacterial lichens was found at the Gössenheim-site and lowest at the Hochtor-site (Table 1). Peer et al. (2010) listed 49 lichen species for the whole Hochtor area. Preliminary results from multi-gene phylogenies indicate that a number of genetically and morphologically distinct taxa had previously been overlooked at several SCIN sites, and several species new to science have been found in the study. Sequences usable as DNA barcodes are produced for all new taxa and for a number of additional species.

**Table 1:** Number of lichen species at all sites

	Öland/S	Gössenheim/G	Hochtor/A	Tabernas/E	Total all sites
All lichens	52	25	**62**	55	144
Chlorolichens	43	18	**51**	41	114
Cyanolichens	9 (17 %)	7 (28 **%**)	10 (16 %)	**14** (25 %)	30 (21 %)

Highest numbers in bold

The Öland and the Gössenheim sites had the highest number of shared species, while the Hochtor and the Tabernas sites seem to be disparate with only 4 similar species (Table 2). The lichen *Psora decipiens* was the only species found at all four sites. Lichen species that were found at three of the four sites were *T. sedifolia* (not at Hochtor), *Cetraria islandica* (not at Tabernas), *Diploschistes muscorum* (not at Tabernas), *Collema tenax* (not at Hochtor), and *Peltigera rufescens* (not found at Tabernas; Tables 1 and 2).

**Table 2:** Number of lichen species shared between sites

	Hochtor/A	Öland/S	Gössenheim/G
Tabernas/E	4/3.5 %	7/7 %	5/6.7 %
Gössenheim/G	7/8.8 %	20/35.1 %	–
Öland/S	18/18.8 %	–	–

### Bryophyte diversity

A list of bryophytes is only available for the alpine Hochtor site (Peer et al. 2010). These authors report 38 bryophyte species from the larger Hochtor area, the majority being mosses with only a few liverworts. Our own analyses of the bryophytes of all sites are still in progress and the data will be provided elsewhere.

### Adaptation/acclimation of key organisms

Key organisms were defined to be those species that occur at all the sites or are at least shared within most of them, as for example the lichen species *Psora decipiens*. First results on the morphology of this lichen show that thallus size differs considerably between the different investigation sites, with the smallest individuals occurring at the southernmost site (Tabernas) with 0.14 ± 0.06 cm^2^ and the largest at the northernmost site (Öland) with 0.78 ± 0.2 cm^2^ (n = 30 independent thalli for each site). Preliminary molecular results indicate that the genotypes of *P. decipiens* are different at the four sites.

### Net primary productivity of crust types

**Fig. 7:**
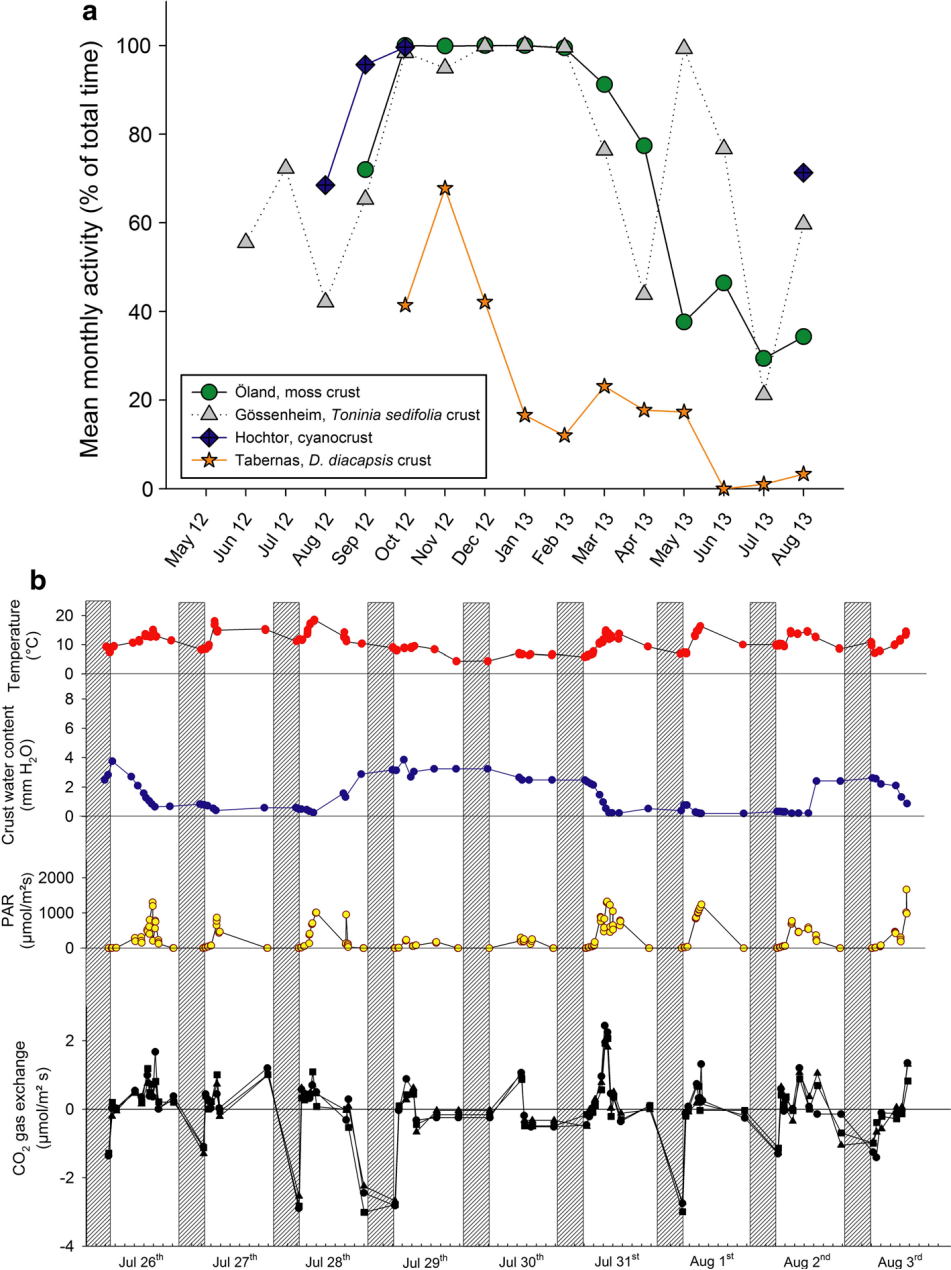
**a** Year round activity (2012–2013) monitoring at all sites: the moss-dominated crust (Öland), the *Toninia sedifolia*-dominated crust (Gössenheim), the cyanobacteria-dominated crust (Hochtor, due to breakage by heavy snow cover, data between October 2012 and July 2013 were lost, monitoring continues for one more year) and the *Diploschistes diacapsis*-dominated crust (Tabernas). **b** Diurnal CO_2_-gas exchange measurements of the cyanobacteria-dominated crust under natural conditions at Hochtor from July 26th to August 3rd 2012; from top to bottom: air temperature 1 m above ground; crust water content expressed as mm precipitation equivalent; ambient light intensity; CO_2_-gas exchange, positive = net photosynthesis, negative = respiration

Annual productivity is obtained by cross-calibrating the field activity measured by chlorophyll fluorescence with the field CO_2_-exchange data. This is done by detecting typical daily patterns of fluorescence and CO_2_ exchange. The end product is the annual carbon balance of BSCs at the four sites and an assessment of the factors that control it (Raggio et al. 2014). First results show that activity periods differ considerably between the four sites (Fig. 7a). A 9 day summary of CO_2_-gas-exchange of the cyanobacteria dominated crust at the alpine Hochtor site in August 2012 showed that this crust type was active in early August (Fig. 7b) and that there was a good correlation between water availability (mm), light (PPFD), temperature (°C) and the resulting CO_2_-gas-exchange. A number of reports of typical soil crust lichen response curves of CO_2_-gas-exchange to water content, light, and temperature as well as diurnal courses have been published and our results are well in accordance with those results (e.g. Hahn et al. 1989; Hahn 1992; Lange et al. 1996, 1997, 1998; Lange 2000; Büdel et al. 2013). Maximal rates of area based net photosynthesis of BSCs from different regions of the world range from 0.11 to 11.5 μmol CO_2_/m^2^ s (Lange 2003) and with about 2.5 μmol CO2/m^2^ s the crusts investigated here are in the lower range of those crusts listed by Lange (2003) that originated from all over the world.

## Conclusions

Species diversity assessments of BSCs show far higher species numbers for the two natural sites, Tabernas, Spain and Hochtor, alpine Austria, compared to the two semi-natural sites at Öland, Sweden and Gössenheim, Germany. However, it is not clear yet if human impact is the major factor for differences in diversity, it could also be other factors such as water availability, soil properties or as yet unknown factors. This however, we can hopefully address after having completed all the data gathering and experimental work. The first results suggest a unique BSC bacterial community at each site and this apparently holds true also for the other organism groups such as lichens and cyanobacteria. The relationships between the variables; crust coverage, diversity, activity, biomass and the water availability at each site, seem to play a major role and needs to be analyzed carefully. Concepts we intend to develop for sustainable management of the two semi-natural and the protection of the two natural sites need to be based on proper knowledge regarding the factors that determine their uniqueness. For example, we cannot begin to guess the recovery times of heavily or slightly disturbed BSCs before the recovery experiments are completed and the specific carbon gain rates are calculated for each site. The initial data and analyses presented here already point out the importance of BSC protection and that the development of appropriate ways to manage biodiversity of BSCs along the latitudinal and altitudinal gradient are essential.

## Electronic supplementary material

Below is the link to the electronic supplementary material.

Fig. 1 Flow chart of the SCIN-project with single work packages and integration levels Supplementary material 1 (JPEG 2460 kb)

Fig. 2 Investigation sites and their equipment. a) Fenced Hochtor site with installed equipment, Austria; b) recovery experiment at Gössenheim, Germany; c) fenced Gössenheim site with chlorophyll fluorescence probes outspread over the area; d) central unit of the MONI-DA; e) explanation of a single MONI-DA-probe. Supplementary material 2 (JPEG 1316 kb)
