# Supplementary figures and images for: Improved appreciation of the functioning and importance of biological soil crusts in Europe: the Soil Crust International Project (SCIN)
Source: Biodivers Conserv. 2014 Mar 2;23(7):1639–58. doi: 10.1007/s10531-014-0645-2 (PMC4058319; doi:10.1007/s10531-014-0645-2)

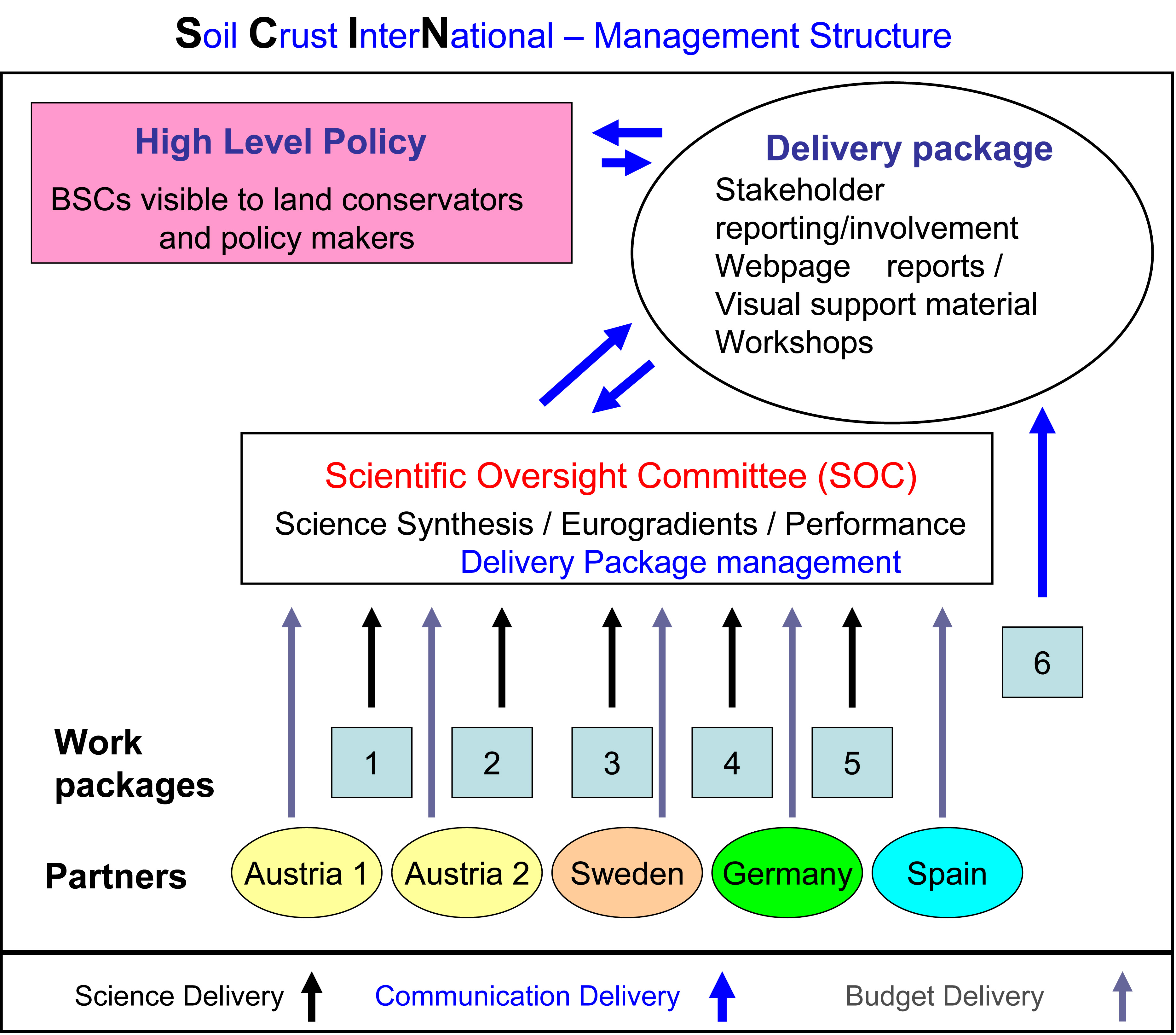

Supplement: Supplementary file 1 — Flow chart of the SCIN-project with single work packages and integration levels Supplementary material 1 (JPEG 2460 kb) [file 10531_2014_645_MOESM1_ESM.jpg]

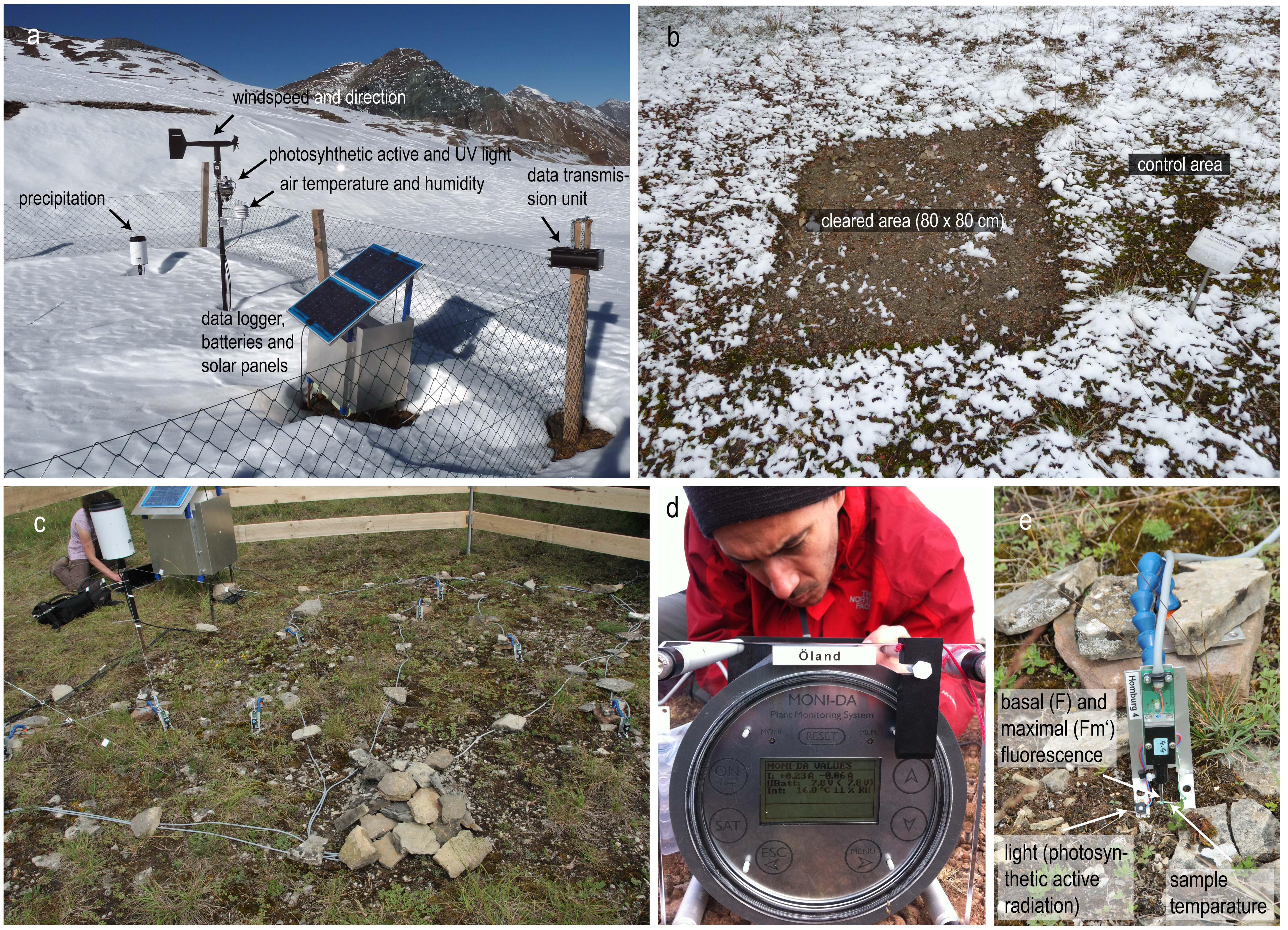

Supplement: Supplementary file 2 — Investigation sites and their equipment. a) Fenced Hochtor site with installed equipment, Austria; b) recovery experiment at Gössenheim, Germany; c) fenced Gössenheim site with chlorophyll fluorescence probes outspread over the area; d) central unit of the MONI-DA; e) explanation of a single MONI-DA-probe. Supplementary material 2 (JPEG 1316 kb) [file 10531_2014_645_MOESM2_ESM.jpg]
